# Supplementary material for: Rapid Limit Test of Eight Quinolone Residues in Food Based on TLC-SERS, a New Limit Test Method
Source: Molecules. 2023 Sep 6;28(18):6473. doi: 10.3390/molecules28186473 (PMC10537116; doi:10.3390/molecules28186473)
Supplement: Supplementary file 1 [file molecules-28-06473-s001.zip › molecules-2577604-supplementary.pdf]

Rapid Limit Test of Eight Quinolone Residues in Food Based on New Limit Test Method: TLC-SERS

Supplementary Materials

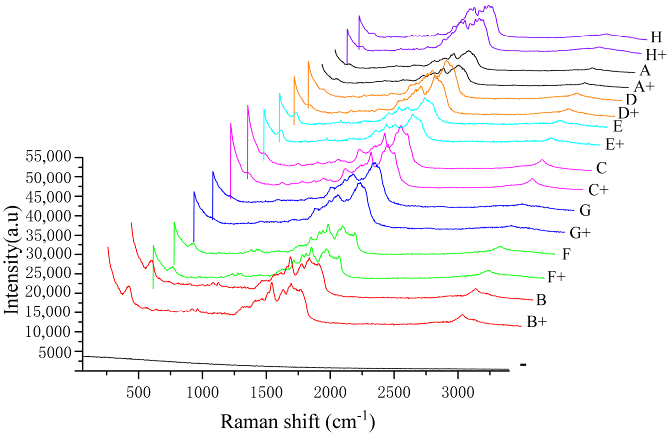

Figure S1. The SERS of the eight quinolones in the simulated positive sample by the TLC-SERS. (H, A, D, E, C, G, F, and B represent Nadifloxacin, Enrofloxacin, Fleroxacin, Sparfloxacin, Ofloxacin,Gatifloxacin, Enoxacin, and Ciprofloxacin, respectively. H^{+}, A^{+}, E^{+}, D^{+}, C^{+}, G^{+}, F^{+}, B^{+} represents Simulated positive samples containing H, A, D, E, C, G, F, and B; "–" represents Negative sample.)

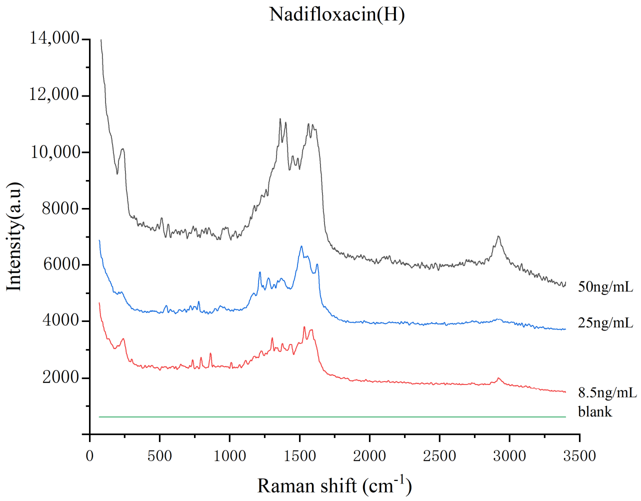

Figure S2. The SERS of the nadifloxacin (H) of different concentrations by the TLC-SERS.

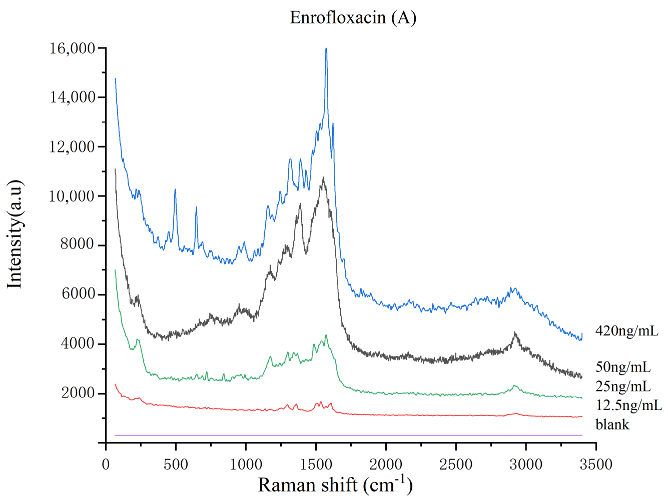

Figure S3. The SERS of the enrofloxacin (A) of different concentrations by the TLC-SERS.

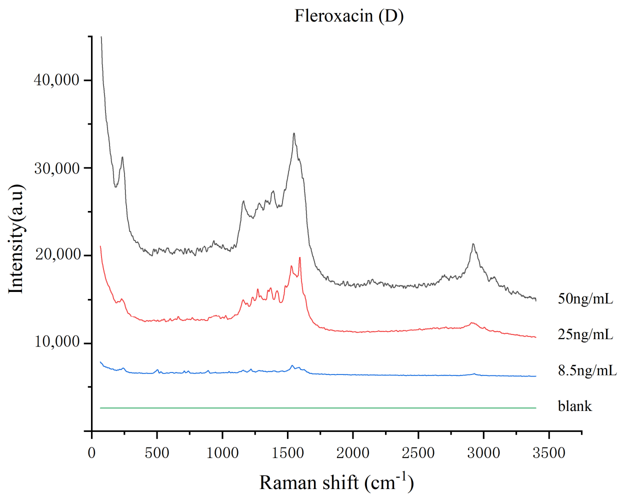

Figure S4. The SERS of the fleroxacin (D) of different concentrations by the TLC-SERS.

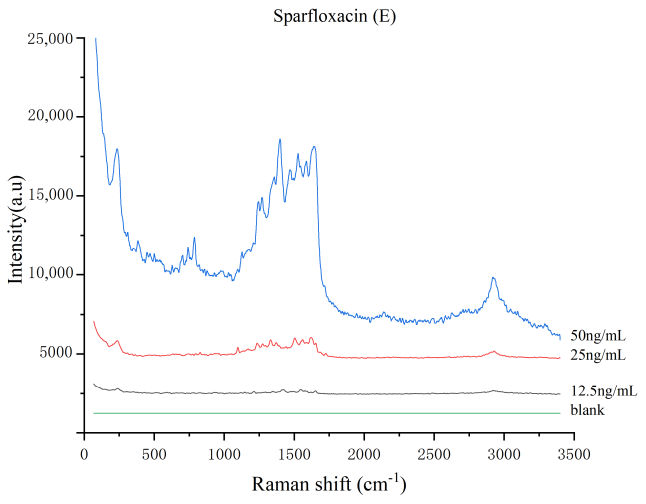

Figure S5. The SERS of the sparfloxacin (E) of different concentrations by the TLC-SERS.

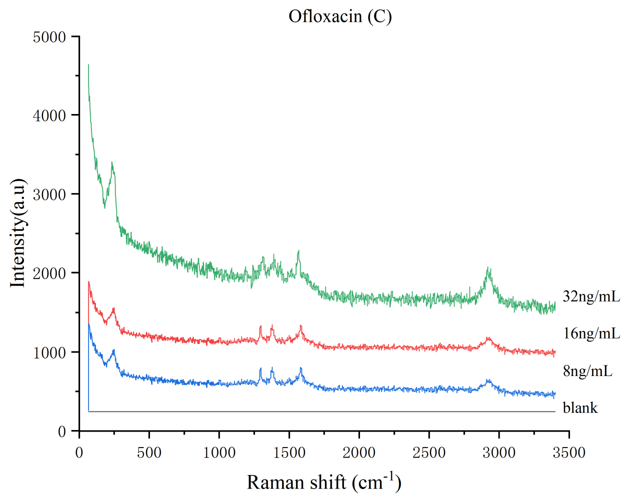

Figure S6. The SERS of the ofloxacin (C) of different concentrations by the TLC-SERS.

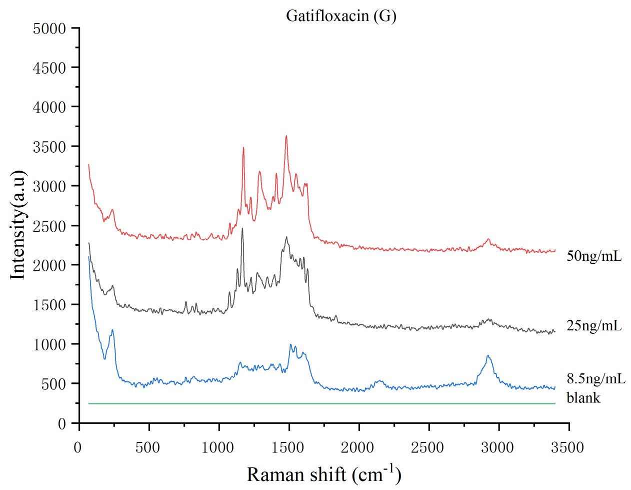

Figure S7. The SERS of the gatifloxacin (G) of different concentrations by the TLC-SERS.

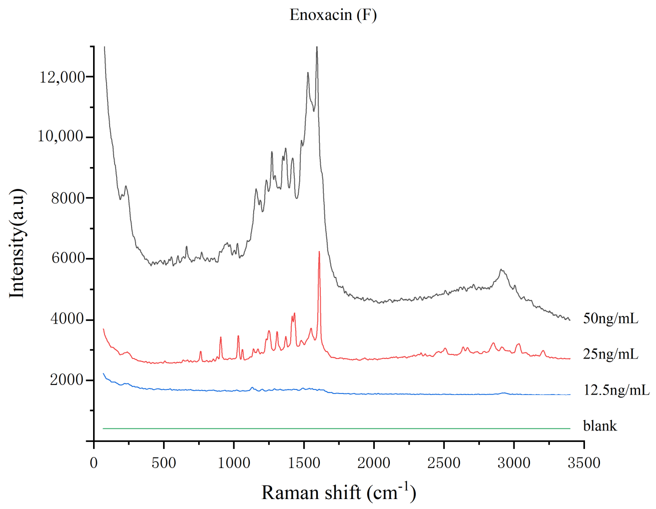

Figure S8. The SERS of the enoxacin (F) of different concentrations by the TLC-SERS.

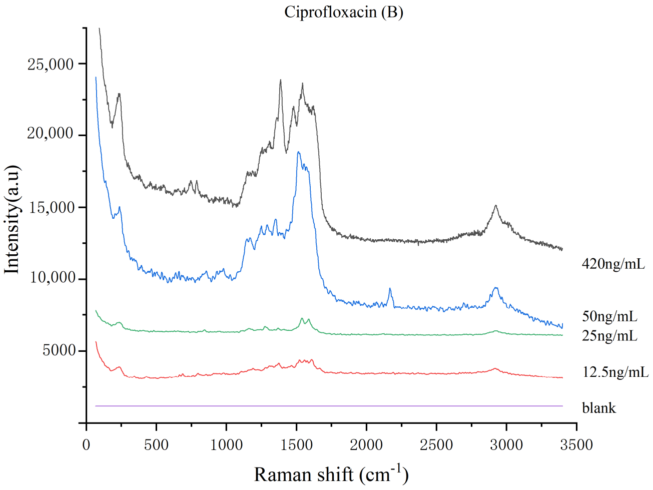

Figure S9. The SERS of the ciprofloxacin (B) of different concentrations by the TLC-SERS.

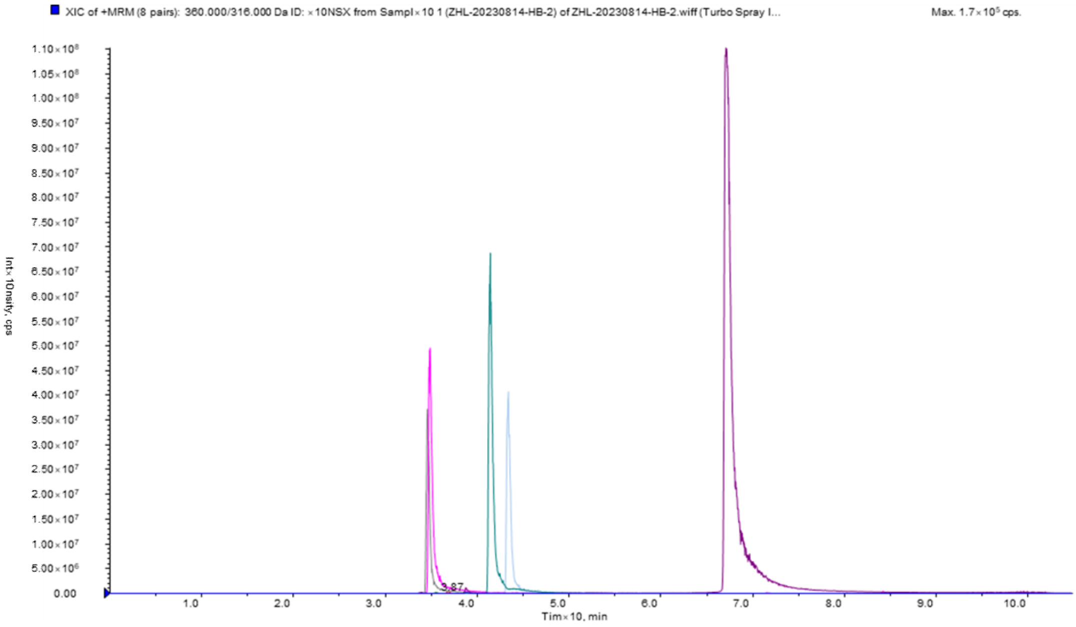

Figure S10. The ion flow chromatograms of eight quinolones.

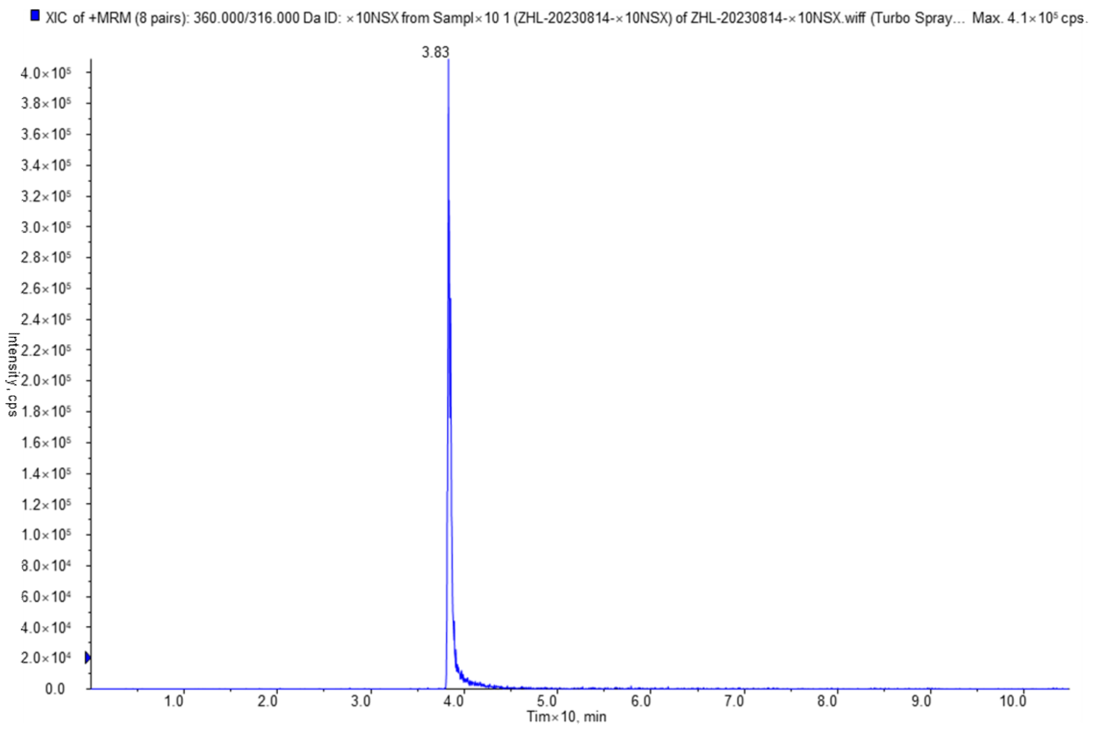

Figure S11. The chromatograms of sample 3 by UPLC-MS/MS.

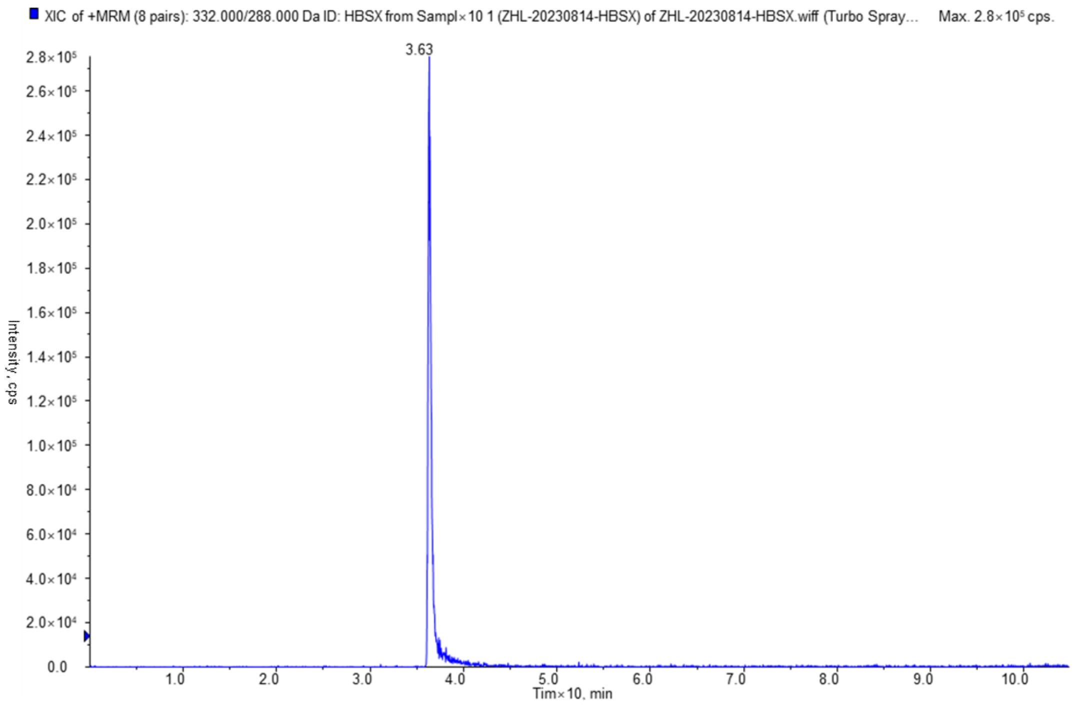

Figure S12. The chromatograms of sample 8 by UPLC-MS/MS.

Table S1. Comparison between the TLC-SERS and the UPLC-MS/MS to determine of the eight quinolones residues in aquatic products.

	UPLC-MS/MS			TLC-SERS		
	LOD_1 (μg/kg)	RSD_1 (%)	Time required (min)	LOD_2 (μg/kg)	RSD_2 (%)	Time required (min)
H	1.0	≦13.1	≧250	2.2	2.9~4.3	≧15
A	1.0	≦15.0	≧250	3.2	2.2~3.8	≧15
D	6.7	≦10.4	≧250	2.2	2.9~4.1	≧15
E	1.0	≦15.0	≧250	4.8	2.9~3.7	≧15
C	1.0	≦15.0	≧250	2.0	3.1~4.9	≧15
G	1.0	≦15.0	≧250	2.2	3.0~4.5	≧15
F	1.0	≦15.0	≧250	4.8	3.1~4.6	≧15
B	1.0	≦15.0	≧250	3.2	2.1~3.9	≧15

Under the UPLC-MS/MS, LOD_1 and RSD_1 are respectively the limit of detection and relative standard deviation Required by the National Standard of the People's Republic of China Announcement No. 1077-1-2008 of the Ministry of Agriculture. Under the TLC-SERS , LOD_2 is the actual limit of detection of the quinolones in the TLC-SERS; RSD_2 is the relative standard deviation of the S/N values at the characteristic peaks（$v_{C\text{-}N}$）from the quinolones reference materials of different concentrations. H, A, D, E, C, G, F, and B represent Nadifloxacin, Enrofloxacin, Fleroxacin, Sparfloxacin, Ofloxacin, Gatifloxacin, Enoxacin, and Ciprofloxacin, respectively.

Table S2. The EF at Raman spectra characteristic peaks of the eight quinolones.

		SERS				Raman				
	Functional group	Raman Shift (cm^{-1})	I_{SERS}	M_{SERS} (μg)	I_{SERS}/M_{SERS}	Raman Shift (cm^{-1})	I_{blank}	M_{blank} (μg)	I_{blank}/M_{blank}	EF
H	$\nu_{C=C}$	1587	12896	2.0×10^{-4}	6.4×10^{7}	1620	562	10	56.2	1.1×10^{6}
	$\nu_{C=C}$	1398	12576	2.0×10^{-4}	6.3×10^{7}	1398	714	10	71.4	8.8×10^{5}
	β_{CH2}, β_{CH3}	1257	8107	2.0×10^{-4}	4.1×10^{7}	1261	155	10	15.5	2.6×10^{6}
	ν_{C-N}	1157	5509	2.0×10^{-4}	2.8×10^{7}	1136	196	10	19.6	1.4×10^{6}
A	$\nu_{C=C}$	1552	8131	4.0×10^{-3}	2.0×10^{6}	1628	655	10	65.5	3.1×10^{4}
	$\nu_{C=C}$	1389	7046	4.0×10^{-3}	1.8×10^{6}	1389	1577	10	157.7	1.1×10^{4}
	β_{CH2}, β_{CH3}	1281	5385	4.0×10^{-3}	1.3×10^{6}	1284	100	10	10.0	1.3×10^{5}
	ν_{C-N}	1173	4539	4.0×10^{-3}	1.1×10^{6}	1178	56	10	5.6	2.0×10^{5}
D	$\nu_{C=C}$	1535	14354	2.0×10^{-4}	7.2×10^{7}	1531	579	10	57.9	1.2×10^{6}
	$\nu_{C=C}$	1379	10803	2.0×10^{-4}	5.4×10^{7}	1381	1994	10	199.4	2.7×10^{5}
	β_{CH2}, β_{CH3}	1298	8285	2.0×10^{-4}	4.1×10^{7}	1296	328	10	32.8	1.2×10^{6}
	ν_{C-N}	1255	6650	2.0×10^{-4}	3.3×10^{7}	1227	520	10	52.0	6.3×10^{5}
E	$\nu_{C=C}$	1549	10418	2.0×10^{-4}	5.2×10^{7}	1533	758	10	75.8	6.9×10^{5}
	$\nu_{C=C}$	1373	7036	2.0×10^{-4}	3.5×10^{7}	1388	1977	10	197.7	1.8×10^{5}
	β_{CH2}, β_{CH3}	1279	7215	2.0×10^{-4}	3.6×10^{7}	1286	3369	10	336.9	1.1×10^{5}
	ν_{C-N}	1176	5145	2.0×10^{-4}	2.6×10^{7}	1176	543	10	54.3	4.8×10^{5}
C	$\nu_{C=C}$	1552	14493	8.0×10^{-5}	1.8×10^{8}	1554	460	10	46.0	4.0×10^{6}
	$\nu_{C=C}$	1394	12124	8.0×10^{-5}	1.5×10^{8}	1400	1488	10	148.8	1.0×10^{6}
	β_{CH2}, β_{CH3}	1300	8055	8.0×10^{-5}	1.0×10^{8}	1300	170	10	17.0	5.9×10^{6}
	ν_{C-N}	1151	6982	8.0×10^{-5}	8.7×10^{7}	1149	287	10	28.7	3.0×10^{6}
G	$\nu_{C=C}$	1562	14826	2.0×10^{-4}	7.4×10^{7}	1556	272	10	27.2	2.7×10^{6}
	$\nu_{C=C}$	1357	11110	2.0×10^{-4}	5.6×10^{7}	1371	232	10	23.2	2.4×10^{6}
	β_{CH2}, β_{CH3}	1286	9437	2.0×10^{-4}	4.7×10^{7}	1280	154	10	15.4	3.1×10^{6}
	ν_{C-N}	1151	7176	2.0×10^{-4}	3.6×10^{7}	1155	140	10	14.0	2.6×10^{6}
F	$\nu_{C=C}$	1540	8635	2.0×10^{-4}	4.3×10^{7}	1547	376	10	37.6	1.1×10^{6}
	$\nu_{C=C}$	1414	9081	2.0×10^{-4}	4.5×10^{7}	1404	4325	10	432.5	1.0×10^{5}
	β_{CH2}, β_{CH3}	1265	5423	2.0×10^{-4}	2.7×10^{7}	1290	679	10	67.9	4.0×10^{5}
	ν_{C-N}	1161	2942	2.0×10^{-4}	1.5×10^{7}	1153	144	10	14.4	1.0×10^{6}
B	$\nu_{C=C}$	1543	11656	4.0×10^{-3}	2.9×10^{6}	1570	414	10	41.4	7.0×10^{4}
	$\nu_{C=C}$	1386	11892	4.0×10^{-3}	3.0×10^{6}	1387	3036	10	303.6	9.9×10^{3}
	β_{CH2}, β_{CH3}	1307	7613	4.0×10^{-3}	1.9×10^{6}	1313	332	10	33.2	5.7×10^{4}
	ν_{C-N}	1151	5472	4.0×10^{-3}	1.4×10^{6}	1162	145	10	14.5	9.6×10^{4}

EF is the Raman enhancement factor, I_{sers} and I_{blank} are the Raman intensities at characteristic peaks of the SERS active substrate and blank substrate; M_{sers} and M_{blank} are the mass (μg) of the quinolones on the SERS active substrate and blank substrate. H, A, D, E, C, G, F, and B represent Nadifloxacin, Enrofloxacin, Fleroxacin, Sparfloxacin, Ofloxacin, Gatifloxacin, Enoxacin, and Ciprofloxacin, respectively.
